# Response to vaccination and immune status after intrauterine exposure to immunomodulating drugs for maternal IBD

**DOI:** 10.1038/s41541-026-01499-5

**Published:** 2026-06-05

**Authors:** Jantien W. Wieringa, Mirjam J. Esser, Matthijs D. Kruizinga, Gerco den Hartog, Lyanne W. Rövekamp, Esther. G. J. Rijntjes-Jacobs, Herbert M. van Wering, Gerdien A. Tramper-Stranders, Willem A. Dik, Marjan Kuijer, Nynke Y. Rots, Gertjan J. A. Driessen

**Affiliations:** 1 Department of Pediatrics, Haaglanden Medical Center, The Hague, The Netherlands; 2 Department of Pediatrics, Division of Paediatric Infectious Diseases and Immunology, Erasmus MC University Medical Center- Sophia Children’s Hospital, Rotterdam, The Netherlands; 3 Department of Pediatrics, Maastricht University Medical Center, MosaKids Children’s Hospital, Maastricht, The Netherlands; 4 Research Institute for Oncology and Reproduction (GROW), Maastricht University, Maastricht, The Netherlands; 5 Department of Pediatrics, Haga Teaching Hospital, Juliana Children’s Hospital, The Hague, The Netherlands; 6 Centre for Infectious Disease Control, National Institute for Public Health and the Environment (RIVM), Bilthoven, The Netherlands; 7 Laboratory of Medical Immunology, Radboud University Medical Center, Nijmegen, The Netherlands; 8 Department of Pediatrics, Division of Neonatology, Leiden University Medical Center, Leiden, The Netherlands; 9 Department of Pediatrics, Amphia Hospital, Breda, The Netherlands; 10 Department of Pediatrics, Franciscus Gasthuis & Vlietland, Rotterdam, The Netherlands; 11 Laboratory Medical Immunology, Department of Immunology, Erasmus MC, University Medical Center, Rotterdam, The Netherlands

**Keywords:** Diseases, Immunology, Medical research

## Abstract

Guidelines on inflammatory bowel disease (IBD) recommend continuing immunomodulating drugs during pregnancy, but their effects on the infant immune system remain uncertain. In this observational study, infants exposed to maternal IBD medication were vaccinated according to the Dutch National Immunization Program. We measured vaccine-specific IgG at 6 and 12 months and leukocytes, lymphocyte subsets, immunoglobulins, and infections at 2, 6, and 12 months of age. Vaccine responses and lymphocyte subsets were compared with historical cohorts and other outcomes with age-specific reference ranges. Vaccine responses following a 3 + 1 vaccination schedule were comparable to or higher than controls following a 2 + 1 schedule for all vaccine components, except filamentous haemagglutinin (FHA) and pneumococcal serotype 19 F. Neutropenia occurred more frequently than expected from reference values, but was self-limiting in all cases. The proportion of infants with T- or total B-cell lymphopenia did not differ from healthy controls. At 6 months, 9.8% of infants had mildly reduced total IgG concentrations, and low IgA was observed in 28.1%, 41.3%, and 15.4% at 2, 6, and 12 months, respectively; significantly more than expected based on reference values. The observed infection rate was similar to that reported in the literature. These findings may help develop clinical follow-up guidelines.

## Introduction

Relapse of inflammatory bowel disease (IBD) during pregnancy is associated with an increased risk of spontaneous abortion, prematurity, and low birth weight^1,2^. To maintain remission, international guidelines recommend continuation of immunomodulating drugs such as thiopurines, adalimumab, infliximab, ustekinumab, and vedolizumab during pregnancy^3,4^. These drugs cross the placenta but have not been associated with adverse pregnancy outcomes or congenital malformations^3,4^. However, their effects on the developing immune system of the infants remain uncertain. Adalimumab, infliximab (both anti-tumor necrosis factor alpha (anti-TNFα) inhibitors), ustekinumab, and vedolizumab are immunoglobulin G1 (IgG1) immunoglobulins that are actively transported across the placenta via the neonatal Fc receptor^5–7^, resulting in high neonatal concentrations often exceeding maternal levels^5,8–11^. Exposure extends beyond the neonatal period because of the long half-life of these biologicals in infants^11^. Anti-TNFα has been detected up to 12 months after birth^11,12^. Concerns arose after five deaths following Bacillus Calmette-Guérin (BCG) vaccination in infants with intrauterine exposure to anti-TNFα^13,14^. Furthermore, neutropenia^15,16^, hypogammaglobulinemia^17^, subtle alterations in T- and B-cell subsets^16,18^, and a reduced mycobacterial response^16^ in infants exposed to anti-TNFα during pregnancy have been described. Although the risk of severe infections does not seem to be increased after intrauterine exposure to anti-TNFα, vedolizumab, or ustekinumab monotherapy^2,19–22^, some studies found an increased risk after combination therapy^11,23^. In addition to safety concerns, studies in adults and children treated with immunomodulating drugs have indicated that vaccine responses might be impaired^24–26^. A reduced response to some components of maternal tetanus, diphtheria, and acellular pertussis (Tdap) vaccine has also been suggested in women who received a Tdap vaccine during pregnancy while being treated with immunomodulating drugs^27,28^.

Findings from small studies on vaccination in infants with intrauterine exposure to immunomodulating drugs have been inconclusive. Responses did not differ between infants exposed to anti-TNFα and infants exposed to other immunomodulating drugs^29,30^, but were lower than historically reported in the general population^30^. In addition, other studies have reported low vaccine responses in some children^17,31^.

In this prospective cohort study, we evaluated whether Dutch National Immunization Program (NIP) vaccines elicit comparable responses in infants with and without intrauterine exposure to immunomodulating drugs and additionally assessed other immunological parameters.

## Results

### Study population

**Fig. 1:**
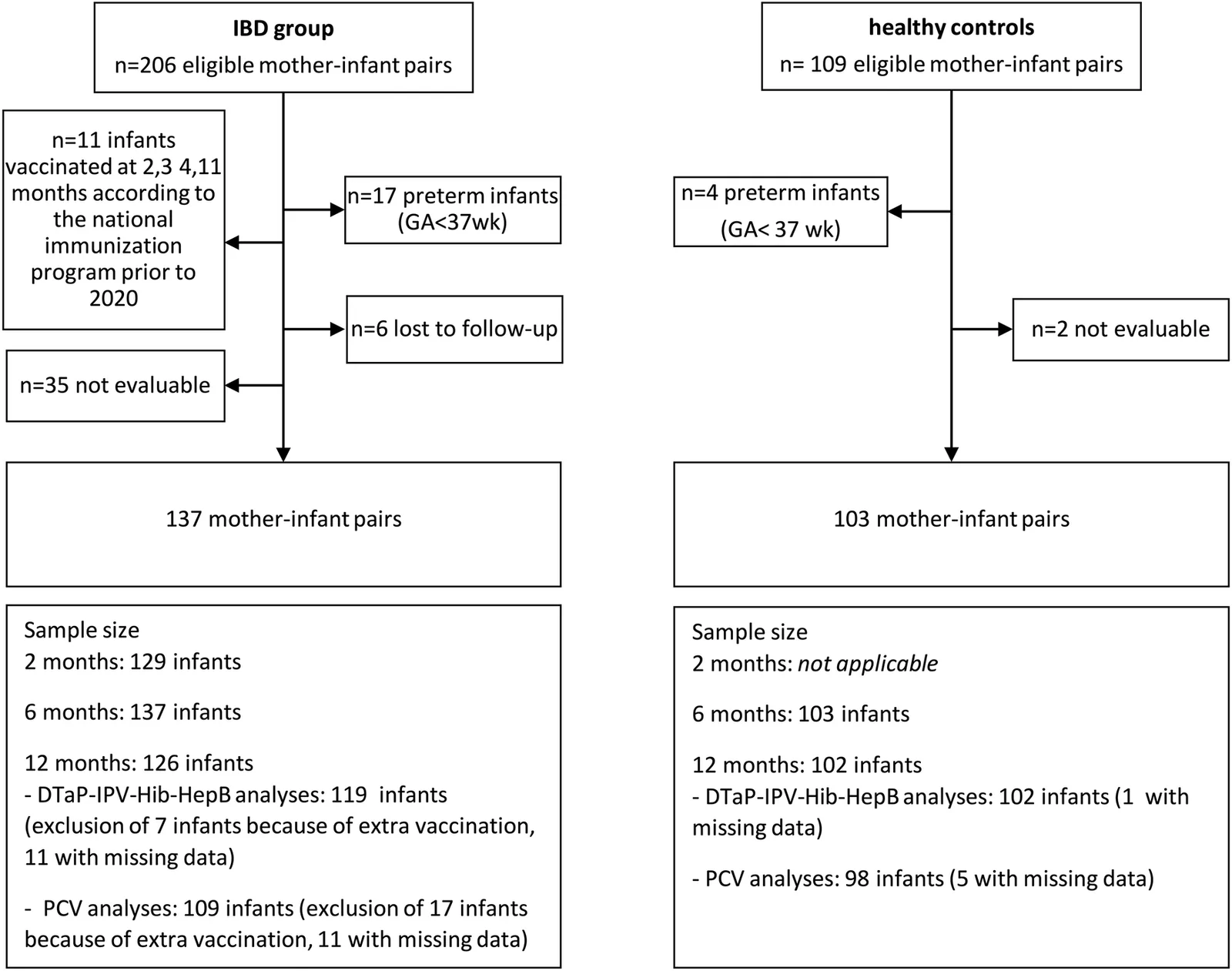
Study flowchart. IBD inflammatory bowel disease, GA gestational age, wk weeks, DTaP5-IPV-Hib-HepB Diphtheria-Tetanus-acellular pertussis-inactivated poliovirus-H influenza type b-Hepatitis B, PCV pneumococcal conjugate vaccine.

For the evaluation of the response to vaccination, data were available for 137 IBD mother-infant pairs and 103 control mother-infant pairs (Fig. 1). In 23 infants (16.8%) in the IBD cohort, an additional 7-month vaccination was administered (10-valent pneumococcal conjugate vaccine [PCV10] in 16 infants, diphtheria, tetanus, 5-component acellular pertussis, inactivated poliovirus, *Haemophilus influenza* type b, hepatitis B [DTaP5-IPV-Hib-HepB] in 6 infants, and both vaccines in 1 infant) because the response to primary vaccination, as measured at some of the participating hospital, was considered insufficient. These infants were excluded from the corresponding analyses at 12 months. The gestational age (GA) at birth, GA at time of maternal Tdap vaccination, and age at 2-month sampling were lower in the IBD group than in controls, while the age at 6- and 12-month sampling and time between vaccination and sampling were higher (Table 1). The percentage of mothers with a Tdap vaccination during pregnancy was significantly lower in the control group compared to the IBD group due to the study design of the control group. Among the IBD mothers, 69 (50.4%) used anti-TNFα, 47 (34.3%) other biologicals, and 21 (15.3%) non-biologicals as primary medication. In total, 22 mothers used a corticosteroid as primary or concomitant medication, mostly until delivery (Supplementary Table 1). The concentrations of biologicals at birth in infants of IBD mothers exceeded the maternal concentration, except for vedoluzimab. Concentrations declined over time and were undetectable in all infants at 12 months of age (Supplementary Table 2).

**Table 1:** Baseline characteristics of IBD mother-infant pairs and healthy control mother-infant pairs^a^

	Healthy controls	IBD cohort any drugs^b^	IBD cohort TNFα^c^	*p*-value Any vs healthy	*p*-value TNFα vs healthy
	*n* = 103	*n* = 137	*n* = 69		
**Mother**					
Age (yr) at delivery, median (IQR)	32.0 (30.0– 34.0)	31.0 (29.0–34.0)	31.0 (29.0– 34.0)	0.26	0.32
IBD duration (yr), median (IQR)	n/a	9.0 (5.3–12.0)	10.0 (5.0– 12.0)	n/a	n/a
IBD type, n (%)					
Crohn	n/a	98 (71.5)	54 (78.3)	n/a	n/a
Ulcerative Colitis	n/a	37 (27.0)	15 (21.7)	n/a	n/a
Undifferentiated	n/a	2 (1.5)	0 (0)	n/a	n/a
Tdap vaccination during pregnancy, n (%)	53 (51.5)	119 (86.9)	57 (82.6)	< 0.001	< 0.001
GA (wk) at Tdap vaccination, median (IQR)	31.0 (30.3– 31.6)	24.0 (23.0–28.0)	24.0 (23.0– 30.0)	< 0.001	< 0.001
**Infant**					
GA (wk) at delivery, median (IQR)	40.3 (39.3– 41.0)	39.3 (38.3–40.6)	39.3 (38.6– 40.6)	< 0.001	0.002
Sex, male, n (%)	41 (39.8)	69 (50.4)	32 (46.4)	0.12	0.43
Part of twins, n (%)	0 (0)	4 (2.9)	4 (5.8)	0.14	0.03
Birth weight (kg), median (IQR)	3.4 (3.1–3.8)	3.3 (3.0–3.7)	3.4 (3.1–3.7)	0.22	0.69
Age(d) at 2mo blood draw, median (IQR)	61.0 (60.0– 62.0)	53.0 (48.0–57.5)	54.0 (48.5– 59.5)	< 0.001	< 0.001
Age(mo) at 6mo blood draw, median (IQR)	6.0 (5.9–6.1)	6.3 (6.0–6.5)	6.2 (6.0–6.5)	< 0.001	< 0.001
Age(mo) at 12mo blood draw, median (IQR)	12.0 (11.9– 12.1)	12.2 (12.1–12.6)	12.2 (12.0– 12.5)	< 0.001	< 0.001
Time (d) between 5mo vaccination and 6mo blood draw, median (IQR)	30.0 (28.0– 33.0)	32.0 (28.0–37.5)	32.0 (27.5– 38.0)	0.01	0.06
Time (d) between 11mo vaccination and 12mo blood draw, median (IQR)	28.0 (28.0– 32.0)	31.0 (26.8–36.0)	30.0 (26.0– 37.0)	0.04	0.15
**Medication mother**					
**Main immunomodulating medication,** ***n*** **(%)**					
*Anti-TNFα*	n/a	69 (50.4)	69 (50.4)	n/a	n/a
Adalimumab	n/a	34 (24.8)	34 (24.8)	n/a	n/a
Infliximab	n/a	34 (24.8)	34 (24.8)	n/a	n/a
Golimumab	n/a	1 (0.7)	1 (0.7)	n/a	n/a
*Other biologicals*	n/a	47 (34.3)	n/a	n/a	n/a
Vedolizumab	n/a	23 (16.8)	n/a	n/a	n/a
Ustekinumab	n/a	24 (17.5)	n/a	n/a	n/a
*Non-biologicals*	n/a	21 (15.3)	n/a	n/a	n/a
Thiopurine^d^	n/a	15 (10.9)	n/a	n/a	n/a
Corticosteroid^e^	n/a	5 (3.6)	n/a	n/a	n/a
Tacrolimus	n/a	1 (0.7)	n/a	n/a	n/a
**Concomitant medication, n (%)**					
*Other biologicals*	n/a	1 (0.7)	n/a	n/a	n/a
Vedolizumab	n/a	1	n/a	n/a	n/a
*Non-biologicals*	n/a	45 (32.8)	n/a	n/a	n/a
Thiopurine^d^	n/a	18	n/a	n/a	n/a
Corticosteroid^e^	n/a	18	n/a	n/a	n/a
5-Aminosalicylate^f^	n/a	14	n/a	n/a	n/a
*None*	n/a	91 (66.4)	n/a	n/a	n/a
**GA (wk) at time of medication cessation, median (IQR)**					
Adalimumab	n/a	34.0 (31.5–38.0)	34.0 (31.5– 38.0)	n/a	n/a
Infliximab	n/a	31.0 (24.0–33.0)	31.0 (24.0– 33.0)	n/a	n/a
Vedolizumab	n/a	32.0 (29.3–34.8)	n/a	n/a	n/a
Ustekinumab	n/a	33.0 (31.0–35.0)	n/a	n/a	n/a
Thiopurine^d^	n/a	39.0 (38.0–40.0)	n/a	n/a	n/a
Corticosteroids^e^	n/a	38.0 (37.0–39.0)	n/a	n/a	n/a
Tacrolimus	n/a	40 0/7 (n/a)	n/a	n/a	n/a

^a^Baseline characteristics of 137 IBD mother-infant pairs and 103 healthy control mother-infant pairs from a historical cohort. ^b^Any drugs = use of any immunomodulating drugs. ^c^TNFα = use of anti-TNFα

^d^Thiopurines: Azathioprine, 6-mercaptopurine, or thioguanine.

^e^Corticosteroids: Prednisolone or budesonide.

^f^5-Aminosalicylate: Mesalazine or sulfasalazine. *Yr* years, *mo* months, *d* days, *IQR* interquartile range, *IBD* inflammatory bowel disease, *anti-TNFα* anti-Tumor Necrosis Factor alpha, *GA* gestational age, *wk* weeks, *Tdap* tetanus, diphtheria, and acellular pertussis vaccination, *kg* kilograms, *d* days, *n/a* not applicable.

Baseline characteristics between Tdap-vaccinated and Tdap-unvaccinated IBD mother-infant pairs were comparable, with the exception of infant’s age at the 2-month vaccination (data not shown). Infants born to Tdap-unvaccinated mothers were slightly older at the 2-month vaccination than infants born to Tdap-vaccinated mothers (63.5 (interquartile range [IQR] 59.0–68.5) vs. 61.0 (57.0–63.0) days, respectively, *p* = 0.02). For T- and B-cell subset analyses, baseline characteristics between infants of IBD mothers and 50 healthy controls were comparable (Supplementary Table 3).

### Proportion of infants with protective vaccine-specific antibody concentrations

For most vaccine components, the proportions of infants with IgG concentrations above protective levels were comparable between exposed and control infants (Tables 2, 3).

**Table 2:** Proportion of infants with protective antibody concentrations against Dt, Tt, Hib PRP, HBsAg, and IPV in infants with intrauterine exposure to immunomodulating drugs compared to healthy controls^a^

Antigen^b^	Healthy controls	IBD any drugs	IBD TNFα	*p*-value healthy vs any	*p*-value healthy vs TNFα
**Primary series (6 months)**	***n*** **=** **103**	***n*** **=** **137**	***n*** **=** **69**		
Dt	70.9	77.4	85.5	0.32	0.04
Tt	99.0	100.0	100.0	0.89	> 0.99
Hib PRP	61.6	94.9	94.2	< 0.001	< 0.001
HBsAg	n/a	100.0	100.0	n/a	n/a
IPV1	n/a	97.1	95.7	n/a	n/a
IPV2	n/a	90.5	87.0	n/a	n/a
IPV3	n/a	89.1	88.4	n/a	n/a
**Booster vaccination (12 months)**^**c**^	***n*** = **103**	***n*** = **119**	***n*** = **60**		
Dt	99.0	99.2	100.0	> 0.99	> 0.99
Tt	100.0	100.0	100.0	> 0.99	> 0.99
Hib PRP	92.9	92.4	93.3	> 0.99	> 0.99
HBsAg	n/a	100.0	100.0	n/a	n/a
IPV1	n/a	100.0	100.0	n/a	n/a
IPV2	n/a	100.0	100.0	n/a	n/a
IPV3	n/a	100.0	100.0	n/a	n/a

^a^Data are presented as percentages (%). ^b^Protective thresholds were defined for anti-Dt IgG and anti-Tt IgG at 0.1 IU/ml, for HBsAg at 10 IU/ml, for Hib PRP at 0.15 μg /ml after primary series and at 1.0 μg /ml after booster vaccination, for anti-IPV1 at 0.7 IU/mL, for anti-IPV2 at 2.1 IU/mL, and for anti-IPV3 at 0.6 IU/mL. ^c^At 12 months, 7 patients were excluded from the IBD group because of an extra DTaP5-IPV-Hib-HepB vaccination at 7 months. *IBD* inflammatory bowel disease, *anti-TNFα* anti-Tumor Necrosis Factor alpha, *Dt* diphtheria toxoid, *Tt* tetanus toxin, *Hib* PRP, H. influenzae type b, *HBsAg* hepatitis B, *IPV* inactivated polio virus, *n/a* not applicable.

**Table 3:** Proportion of infants with protective antibody concentrations against pneumococcal conjugate vaccine serotypes in infants with intrauterine exposure to immunomodulating drugs compared to healthy controls^a^

Antigen^b^	healthy	IBD any	IBD TNFα	*p*-value healthy vs any	*p*-value healthy vs TNFα
**Primary series (6 months)**	***n*** = **102**	***n*** = **137**	***n*** = **69**		
Ps 1	93.1	95.6	94.2	0.58	> 0.99
Ps 4	70.6	94.9	95.7	< 0.001	< 0.001
Ps 5	90.2	95.6	95.7	0.16	0.30
Ps 6B	51.0	65.0	58.0	0.04	0.45
Ps 7 F	98.0	99.3	98.6	0.80	> 0.99
Ps 9 V	91.2	95.6	92.8	0.26	0.93
Ps 14	95.1	94.2	91.3	0.98	0.50
Ps 18 C	75.6	97.1	97.1	< 0.001	< 0.001
Ps 19 F	93.1	96.4	95.7	0.41	0.72
Ps 23 F	62.8	62.0	59.4	> 0.99	0.78
**Booster vaccination (12 months)**^**c**^	***n*** = **98**	***n*** = **109**	***n*** = **54**		
Ps 1	100.0	100.0	100.0	> 0.99	> 0.99
Ps 4	99.0	99.1	100.0	> 0.99	> 0.99
Ps 5	100.0	100.0	100.0	> 0.99	> 0.99
Ps 6B	100.0	100.0	100.0	> 0.99	> 0.99
Ps 7 F	100.0	100.0	100.0	> 0.99	> 0.99
Ps 9 V	100.0	100.0	100.0	> 0.99	> 0.99
Ps 14	98.0	97.3	94.4	> 0.99	0.35
Ps 18 C	100.0	99.1	100.0	> 0.99	> 0.99
Ps 19 F	100.0	100.0	100.0	> 0.99	> 0.99
Ps 23 F	99.0	98.2	98.2	> 0.99	> 0.99

^a^Data are presented as percentages (%). ^b^Protective threshold was defined for pneumococcal serotypes at 0.35 μg/ml. ^c^At 12 months, 17 patients were excluded from the IBD group because of an extra pneumococcal vaccination at age 7 months. *IBD* inflammatory bowel disease, *anti-TNFα* anti-Tumor Necrosis Factor alpha, *Ps* pneumococcal serotype.

The proportion of infants with protective anti-Hib polyribosylribitol phosphate (PRP) IgG was higher in exposed infants than in controls at 6 months of age (94.9% vs. 61.6%, *p* < 0.001), while at 12 months no difference was observed. All exposed infants had protective anti-hepatitis B surface antigen (HBsAg) concentrations at 6 and 12 months, and anti-IPV1-3 concentrations at 12 months, comparable to reported proportions in the general population^32,33^. At age 6 months, the proportion of infants with protective IgG concentrations was higher in exposed infants than controls for pneumococcal serotypes 4 (94.9% vs. 70.6%, *p* < 0.001) and 18 C (97.1% vs. 75.6%, *p* < 0.001). At 12 months, these proportions were comparable for all serotypes. At 6 months, 99.3% of exposed infants had IgG against ≥ 5 PCV10 serotypes above the protective levels, compared with 93.1% of unexposed infants (*p* = 0.02).

### Geometric mean concentrations of vaccine-specific antibodies

**Fig. 2:**
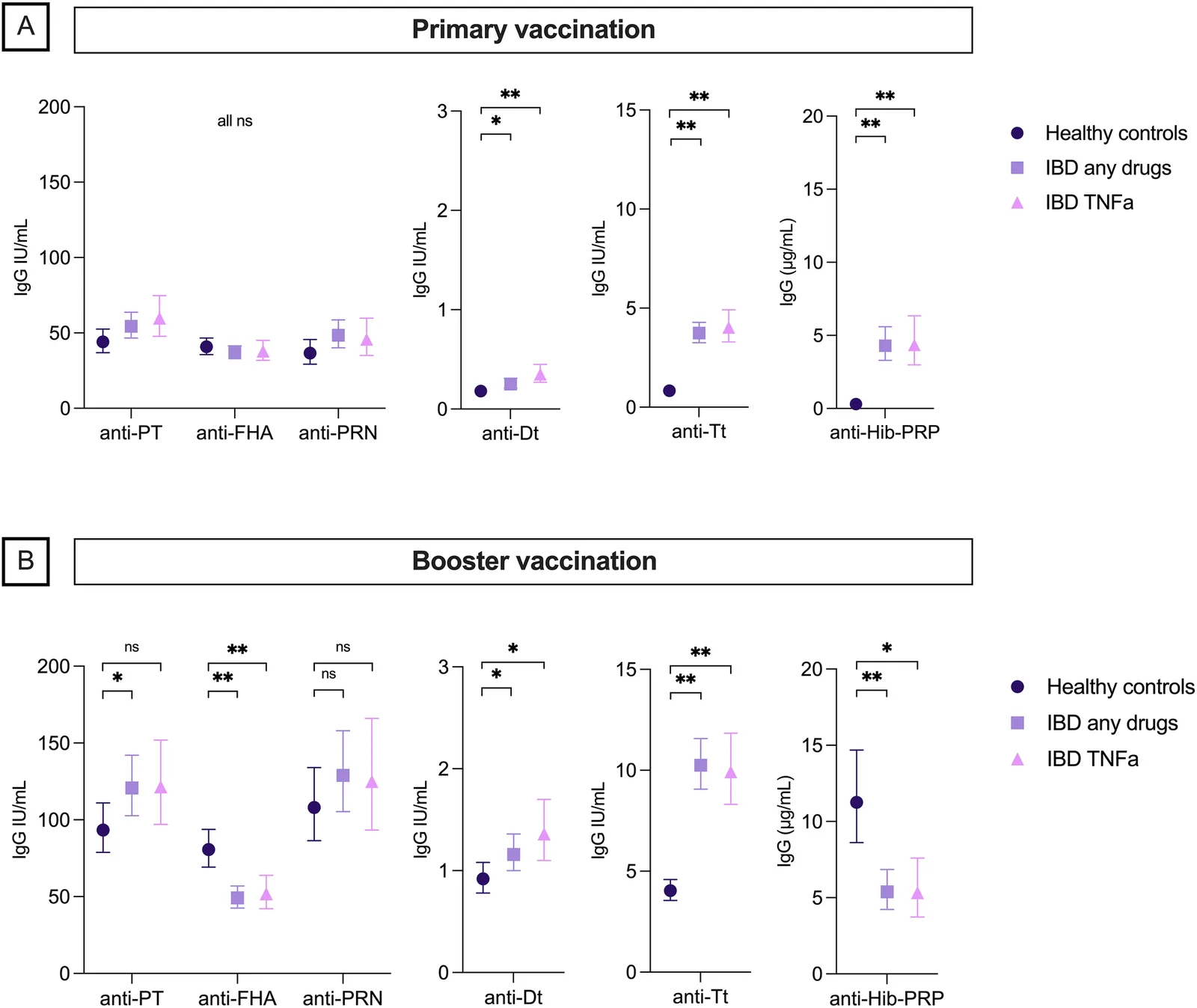
GMCs of IgG antibodies against pertussis vaccine components (PT, FHA, PRN), Dt, Tt, and Hib PRP in infants with intrauterine exposure to immunomodulating drugs and healthy controls. GMCs of IgG antibodies against vaccine components after primary series at 6 months (**A**) and after booster vaccination at 12 months (**B**). Data are presented as geometric means with 95% confidence intervals. *GMC* Geometric mean concentration, *IBD* inflammatory bowel disease, *anti-TNFα* anti-Tumor Necrosis Factor alpha, *PT* pertussis toxin, *FHA* filamentous haemagglutinin, *PRN* pertactin, *Dt* diphtheria toxoid, *Tt* tetanus toxin, *Hib PRP* H. influenzae type b, *ns* non-significant, *p < 0.05, **p < 0.0013.

**Fig. 3:**
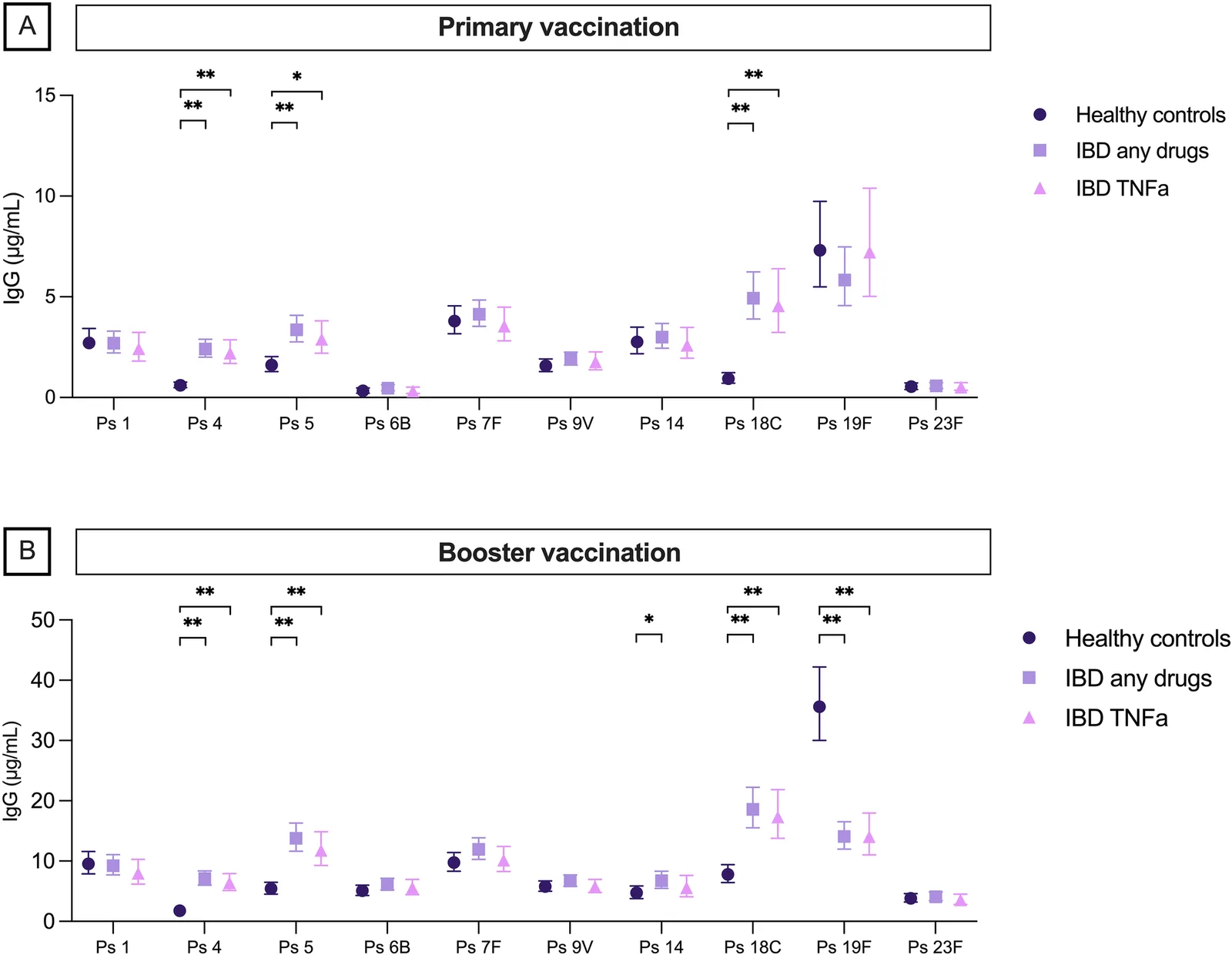
GMCs of IgG antibodies against pneumococcal conjugate vaccine components in infants with intrauterine exposure to immunomodulating drugs and healthy controls. GMCs of IgG antibodies against PCV10 serotypes after primary series at 6 months (**A**) and after booster vaccination at 12 months (**B**). Data are presented as geometric means with 95% confidence intervals. *GMC* Geometric mean concentration, *IBD* inflammatory bowel disease, *anti-TNFα* anti-Tumor Necrosis Factor alpha, *Ps* pneumococcal serotype. *p < 0.05, **p < 0.0013.

For most vaccine components, infants exposed to immunomodulatory drugs had comparable or higher vaccine-specific IgG geometric mean concentrations (GMCs) than unexposed controls (Figs. 2, 3; Supplementary Tables 4 and 5). After primary series (6 months), no differences in antibody concentrations against pertussis antigens (pertussis toxin [PT], filamentous haemagglutinin [FHA], pertactin [PRN]) were observed. After booster vaccination (12 months), exposed infants had lower anti-FHA GMC (49.2 (95% confidence interval [CI] 42.5–56.9) vs. 80.6 (69.3–93.8) IU/mL, *p* < 0.001) and lower anti-Hib GMC (5.38 (4.23–6.85) vs. 11.25 (8.62–14.68) μg/ml, *p* < 0.001), even though anti-Hib GMC was higher at 6 months in exposed infants (4.30 (3.30–5.60) vs. 0.30 (0.22–0.41) μg/ml, *p* < 0.001). At 6 and 12 months, GMCs for pneumococcal serotypes 4, 5, and 18 C were significantly higher in exposed than unexposed infants, whereas serotype 19 F GMC was lower in exposed infants at 12 months (14.08 (11.98–16.55) vs. 35.60 (30.02–42.22) µg/mL, *p* < 0.001).

Sensitivity analyses including infants who received an additional 7-month booster did not significantly alter the results (data not shown).

### Infants exposed to anti-TNFα or other biologicals

Subgroup analyses comparing responses to vaccination between infants exposed to anti-TNFα and controls showed results similar to those observed in the analysis of the total group (Tables 2 and 3; Figs. 2 and 3; Supplementary Table 6 and 7). At 6 months, 98.6% of anti-TNFα exposed infants had IgG against ≥5 PCV10 serotypes above protective levels, compared with 93.1% of unexposed infants (*p* = 0.20).

Subgroup analyses comparing responses to vaccination between infants exposed to other biologicals (vedoluzimab or ustekinumab) and healthy controls showed results mostly consistent with those observed in the total group (data not shown). One exception, the higher proportion with protective IgG concentrations for pneumococcal serotype 4 in the exposed group was no longer statistically significant after correction for multiple testing.

### Infants of Tdap-vaccinated vs Tdap-unvaccinated Mothers

To assess the effect of maternal Tdap vaccination in the IBD cohort, we compared IgG GMCs against all Tdap components between infants of vaccinated and unvaccinated mothers (Supplementary Table 8). At 6 months, GMCs against PT, FHA, and diphtheria toxoid (Dt) were lower in infants of Tdap-vaccinated mothers compared to infants of unvaccinated mothers. At 12 months, no significant differences were observed. In the anti-TNFα group, infants of Tdap-vaccinated mothers had lower GMCs at 6 months of age against PT, FHA, fimbriae types 2 and 3 (FIM2/3), and Dt compared to infants of Tdap-unvaccinated mothers, however, these differences did not remain statistically significant after correction for multiple testing (Supplementary Table 9).

### Leukocyte, lymphocyte, and immunoglobulin concentrations

At 2, 6, and 12 months of age, the median total leukocyte count of exposed infants was within the reference range (Supplementary Table 10). At 12 months, neutropenia occurred more frequently in infants of IBD mothers than expected from reference values (9.8% vs. 2.5%, *p* < 0.001), whereas neutropenia was not present in any of the infants at birth, in 5.1% at 2 months, and in 1.5% at 6 months. One infant had neutrophils < 500/µL at the age of 2 months, which was self-limiting. In all other cases, the neutropenia was mild and also self-limiting. None of the infants developed severe bacterial infections. Monocyte count was below reference values at 2, 6, and 12 months of age in 16.2%, 9.9%, and 10.3% of infants, respectively.

In exposed infants, higher median numbers of natural killer (NK)-, total B-, naïve mature-, and natural effector B cells were observed in cord blood (Supplementary Fig. 1; Supplementary Table 11). At 6 and 12 months of age, no differences in median numbers were observed in any B-cell subset. In contrast, median T-cell numbers were significantly higher at 6 and 12 months in exposed infants compared to controls (4940/µL (IQR 3698–6013) vs. 3420/µL (2180–4300), *p* = 0.002 and 4280/µL (3365–5120) vs. 3365 µL (2688–3620), *p* = 0.008, respectively). The number of infants with T- or B-cell lymphopenia was not significantly higher than the expected 5% (Supplementary Table 12). Memory B cell lymphopenia occurred in 16 infants (13.3%) at birth and 14 infants (11.1%) at 6 months, and this was higher than the expected 5% (*p* < 0.001 and *p* = 0.004, respectively).

Median total IgM, IgA, IgG, and IgG1-4 concentrations in infants born to mothers with IBD were comparable to reference values at 2, 6, and 12 months of age (Supplementary Table 13). At all time points, the proportion of infants with total IgA concentrations below the reference value was higher than expected in the general population (*p* < 0.001). At 6 months, 13 infants (9.8%) had total IgG concentrations below the reference range (ranging from 170 to 240 mg/dL, reference range 260-1390 mg/dL), representing a greater proportion than the expected 2.5% in the general population (*p* < 0.001). At 12 months, this had resolved spontaneously in most infants, and at that time, only 3 (2.4%) still had a mild IgG deficiency (*p* = 0.62).

### Infections

In the first year of life, infants of IBD mothers had a median of 2.0 (IQR 1.0–3.0) infections associated with fever, antibiotic use, or hospitalization. This number of infections seems comparable to the previously reported average of three to four infections in the general infant population^34,35^. Thirty-eight (29%) received ≥ 1 course of antibiotics, and 12 (9.1%) were hospitalized at least once. The most common infections leading to hospitalization were viral respiratory tract infections (Supplementary Table 14). In none of the infants a vaccine-preventable infection was reported.

## Discussion

This multicenter prospective cohort study shows that the responses to vaccination in infants with intrauterine exposure to immunomodulating drugs following a 3 + 1 schedule are comparable to healthy controls following a 2 + 1 schedule for most NIP vaccine components. In addition, we did not find clinically significant or persisting neutropenia, abnormalities in T- or B-cell subsets, or an increased infection rate compared to the general infant population in exposed infants during the first year of life. To our knowledge, this is the largest study to date reporting detailed immunological parameters in infants with intrauterine exposure to immunomodulating drugs.

Previous smaller studies reported a potential negative impact of prenatal exposure to immunomodulating therapy on the developing immune system, including neutropenia^15,16^, hypogammaglobulinemia^17^, subtle alterations in T and B-cell subsets^16,18^, and a reduced response to mycobacteria^16^. Some infants in our cohort developed neutropenia, but unlike previous reports^15,16^, it did not occur directly after birth. All cases resolved spontaneously within weeks without severe infections. In the previously described severe cases, requiring granulocyte colony-stimulating factor (G-CSF), a direct toxic effect of anti-TNFα on the bone marrow or anti-TNFα induced autoantibodies were suggested as causative mechanisms for the occurrence of neutropenia^15,16^. The cause of neutropenia in our cohort remains uncertain. Autoantibody testing was not performed. Although an association with intrauterine exposure to anti-TNFα cannot be excluded, other possible explanations, like post-viral neutropenia, might also be considered.

The most important observation was the reassuring response to active vaccination during the first year of life. At 6 months of age, after primary series, most infants acquired protective antibody concentrations, and GMCs of antibodies in exposed infants were comparable to or higher than those observed in controls. Protective antibody concentrations at this age are critical for protection during the first year of life. Lower GMCs in exposed infants were observed only for FHA, Hib PRP, and pneumococcal serotype 19 F at 12 months. However, almost all infants had protective IgG concentrations for Hib PRP (99.0%) and pneumococcal serotype 19 F (100%) at 12 months. Therefore, the lower GMCs are unlikely to be clinically relevant. For anti-FHA, no correlate of protection exists, but anti-FHA GMC being comparable to controls at 6 months suggests that this finding is also unlikely to have clinical implications.

Given the mode of action, the active placental transfer of biologicals^5–7^, and their long half-life^11,12^, concerns have been raised regarding the vaccine responses in infants exposed to maternal anti-TNFα therapy. In our previous study on the response to Tdap vaccination in mothers treated with anti-TNFα, we observed lower passively transferred anti-pertussis IgGs at birth compared to healthy controls^28^. However, in the present study, the overall response to vaccination, even in the presence of considerable concentrations of anti-TNFα at birth, was reassuring.

We did not observe any major changes in T and B-cell subsets in infants exposed to immunomodulating drugs. Although the proportion of infants with low memory B cells at birth and 6 months was higher than expected based on reference values, this did not seem to affect B-cell function, as reflected by intact vaccine responses. This is in line with another recent study that reported no decrease in total T- and B-cell counts at a median age of 10 weeks^36^. Unexpectedly, we observed higher T-cell counts in infants born to mothers with IBD compared to controls at both 6 and 12 months. The underlying mechanism for this increase remains unclear. Future studies assessing T-cell function could provide further insight into the immunological implications of these findings.

Regarding humoral immunity, more exposed infants than expected based on reference values had low total IgA concentrations at all time points and low total IgG concentrations at 6 months of age. The finding of low IgA concentrations is noteworthy, as IgA deficiency has been associated with an increased risk of IBD^37,38^. It remains unclear whether these low IgA concentrations result from intrauterine exposure to immunomodulating drugs, might reflect a genetic predisposition linked to maternal IBD, or represent physiological hypogammaglobulinemia of infancy, as IgA levels are typically low in early infancy and increase gradually during the first years of life. Unfortunately, we did not have access to maternal IgA or IgG concentrations before the onset of their IBD or before initiation of IBD treatment, limiting our ability to distinguish between these possibilities.

Similarly, the observed low IgG concentrations at 6 months of age need to be interpreted with care. While intrauterine exposure to immunomodulating drugs may contribute, reduced transplacental transfer of maternal IgG might also explain these findings. In addition, subclass analysis of IgG is even more challenging as no well-established reference values for infants in this age group exist. Reassuringly, low total IgG concentrations had resolved in most infants by 12 months of age.

The median number of two infections during the first year of life observed in infants exposed to biologicals in our cohort appears comparable to the previously reported average of three to four infections in the general infant population^34,35^. However, direct comparison is challenging because definitions of infection varied across studies, and differences in country- and epidemic-specific exposure also influence infection rates.

The results should be interpreted with care. The reassuring responses to vaccination occurred in the context of an additional 2-month dose for infants after intrauterine exposure to immunomodulating drugs as part of the Dutch NIP, even if mothers received Tdap vaccination during pregnancy. This undoubtedly influenced vaccination outcomes. The regular Dutch NIP schedule was adapted to a 3-5-11 schedule after maternal Tdap introduction, as high maternal pertussis antibodies persist until at least 3 months of age; this schedule was used in our control cohort^39^. However, the response to pneumococcal vaccination, given at 3, 5, and 11 months in both groups, was also comparable.

In healthy infants, the presence of high concentrations of maternally derived pertussis-specific antibodies following maternal Tdap vaccination can reduce response to active immunization compared to infants of unvaccinated mothers^39^. Similarly, IgG GMCs against most Tdap components at 6 months of age were lower in infants of Tdap-vaccinated versus unvaccinated IBD mothers. However, in infants exposed to anti-TNFα, GMCs did not significantly differ between infants of Tdap-vaccinated and unvaccinated mothers at 6 and 12 months after correction for multiple testing, suggesting minimal interference. Whether exposure to anti-TNFα affects this interference has to be explored in a separate study. Future studies incorporating mother-child paired analyses could provide further insights into maternal antibody transfer and its impact on vaccine responses.

While our study is the largest to date, a few limitations warrant acknowledgment. First, the number of infants exposed to each specific immunomodulating drug remains limited, preventing meaningful subgroup analyses. Likewise, subgroups were too small to perform meaningful correlation analyses between concentrations of maternal medication and the IgG concentrations against different vaccine components or other immunological outcomes. Secondly, some differences between our study cohort and the historical controls existed. Control of vaccination timing was not possible due to the observational study design, resulting in different vaccination and sampling timing. However, by using a linear regression model, we adjusted for timing of vaccination and maternal Tdap vaccination status. We did not adjust for GA at maternal Tdap vaccination. As later vaccination during pregnancy has been associated with higher pre-vaccination antibody concentrations in the infant^40^, and higher pre-vaccination concentrations may result in lower post-vaccination concentrations^39^, this factor may have influenced the results to some extent. Furthermore, the infants exposed to immunomodulating drugs received a slightly different vaccine. Exposed infants were vaccinated with a 5-component pertussis hexavalent vaccine (*Vaxelis)*, compared with a 3-component pertussis hexavalent vaccine *(Infanrix Hexa)* in controls. In addition, nationwide measures to reduce severe acute respiratory syndrome coronavirus 2 (SARS-CoV-2) related infection risk during the IBD cohort study period (2018-2023), but not during the control period (2014–2016), likely have affected maternal IgG boosting. Twenty-three infants (16.8%) in the IBD group with insufficient 6-month serological responses were excluded from the 12-month analyses because they received an additional 7-month booster. Excluding these infants might have influenced the 12-month results. However, sensitivity analyses including these infants yielded similar results, suggesting that their exclusion did not significantly affect the overall findings. Finally, a true evaluation of vaccine effectiveness requires assessment of disease incidence, which is only possible in large prospective studies.

In conclusion, in infants with intrauterine exposure to immunomodulating drugs for maternal IBD, the response to vaccination in the first year of life following a 3 + 1 schedule was comparable to the vaccine response in healthy controls following a 2 + 1 schedule for almost all NIP vaccine components. Self-limiting neutropenia and mild hypogammaglobulinemia occurred more frequently than expected from reference values in the general population. The observed infection rate was similar to that reported in the literature. These findings may help the development of guidelines for the clinical follow-up of these infants. Based on our observations, routing blood screening in the first year of life has limited value in asymptomatic infants exposed to maternal IBD medications. Further studies will be necessary to fully understand the long-term impact of these drugs on the infant immune system.

## Methods

### Study design and setting

For this observational cohort study, pregnant women with IBD treated with immunomodulating drugs able to cross the placenta (adalimumab, infliximab, golimumab, vedolizumab, ustekinumab, thiopurines, corticosteroids, tacrolimus) were prospectively recruited in the PETIT (Pregnancy Exposure to TNF alpha inhibitors and Immunological effect) study cohort at 16 hospitals in the Netherlands between December 2018 and March 2023. The study was approved by the Dutch Medical Ethical Committee Leiden-Den Haag-Delft (NL63910.098.17) and institutional review boards of the Haga Teaching Hospital (The Hague, reference number [ref.] T18-044), Haaglanden Medical Center (The Hague, ref. 2017-106), Erasmus Medical Center (Rotterdam, ref. MEC-2019-0413), Franciscus Gasthuis (Rotterdam, ref. 2020-014), Amphia Hospital (Breda, ref. 1679), and Leiden University Medical Center (Leiden, ref. L20.037). Written informed consent was obtained from all participating women and from the parents or legal guardians of the infants.

### Follow-up and vaccination according to the Dutch NIP

Information on maternal demographics and clinical characteristics was obtained. All women were offered maternal 3-valent Tdap vaccination (*Boostrix)* from the 22^nd^ week of GA onwards according to the Dutch NIP (not part of the study protocol). Clinical data on the infants, including the number of infections that were accompanied by fever, required antibiotics, or hospitalization, were collected at birth and outpatient visits in the first year of life. Blood samples were taken for clinical follow-up of the children at birth and at 2, 6, and 12 months. IgG antibody concentrations against PT, Hib PRP, and vaccine-type pneumococcal capsular polysaccharides (1, 4, 5, 6B, 7 F, 9 V, 14, 18 C, 19 F, and 23 F) were also determined for clinical follow-up (not as part of the study protocol) at the participating hospitals. Concentrations were measured using a LIAISON® CLIA assay (Diasorin, Saluggia, Italy) for PT, the VaccZyme ELISA (The Binding Site, Birmingham, UK) for Hib PRP, and an ISO 15189-accredited, in-house Luminex assay for pneumococcal antibodies. Some of the treating physicians decided to give an additional vaccination at 7 months of age when pertussis, Hib, or pneumococcal serotypes antibody concentrations were interpreted to be low (anti-PT IgG < 10 IU/ml, anti-Hib PRP IgG < 0.15 μg/ml, or if 5 or more anti-pneumococcal serotype antibody concentrations were below 0.35 μg /ml).

According to the Dutch NIP all infants of Tdap-unvaccinated mothers or Tdap-vaccinated mothers with risk factors for reduced transplacental transfer of Tdap antibodies, e.g., immunomodulating therapy during pregnancy, receive an additional vaccination with a 5-antigen pertussis component containing hexavalent combination vaccine (DTaP5-IPV-Hib-HepB) at 2 months of age^41^. All infants of IBD mothers were therefore vaccinated at 2,3,5, and 11 months of age (3 + 1 schedule using *Vaxelis*) (Supplementary Table 15)^42,43^. The PCV10 (*Synflorix)* was administered at 3, 5, and 11 months according to the regular NIP for PCV. Vaccine administration was not part of the study protocol.

### Historical control cohort for comparison of vaccine responses

Responses to vaccination were compared with data from a healthy control group of infants from a previous study on maternal Tdap vaccination (ClinicalTrialsRegister.eu (EudraCT 2013-003090-98) and trialregister.nl (number NTR16010))^39^. These women either received maternal Tdap vaccination during pregnancy at 30–32 weeks of gestation or within 48 hours after delivery^39^. Infants were vaccinated at 3,5, and 11 months of age (2 + 1 schedule) using a 3-antigen pertussis component containing hexavalent vaccine (DTaP3-IPV-Hib-HepB, *Infanrix Hexa*) and PCV10 (*Synflorix)* (Supplementary Table 16). This hexavalent combination vaccine lacks FIM2/3 antigens, whereas the current hexavalent vaccine does contain these antigens. Pregnant women were vaccinated with a Tdap vaccine (*Boostrix)*, without FIM2/3 antigens.

### Historical cohort for comparison of lymphocyte subsets

For the evaluation of the T- and B-cell subsets, results were compared to a Dutch healthy control cohort^44^. Leukocyte and immunoglobulin concentrations were compared to reference values from the literature^45,46^.

### Exclusion criteria

Infants were excluded if (1) they were preterm, (2) not vaccinated according to the current NIP, 3) the 5-month vaccination was postponed for more than 1 month, or (3) blood samples were not taken between 20 and 50 days after vaccination. Infants who received an additional vaccination at 7 months of age were excluded from the corresponding analysis at 12 months of age.

### Blood sampling and analysis

Blood samples were taken at birth (maternal blood and cord blood), and in the infant at 2 months (preceding active immunization), 6 months (20–50 days after the primary immunization series), and 12 months (20–50 days after the booster immunization).

### Measurement of biological concentrations

Concentrations of adalimumab, infliximab, golimumab, ustekinumab, and vedolizumab in IBD mother-infant pairs were measured using in-house developed ELISAs by Sanquin Diagnostic Services as previously described in ref. ^47–49^. The lower limits of detection (LLOD) of these ELISAs were 0.03 µg/ml for infliximab, 0.01 µg/ml for adalimumab, 0.005 µg/ml for golimumab, 0.005 µg/ml for ustekinumab, and 0.01 µg/ml for vedolizumab.

### Immunophenotyping of lymphocytes

Total numbers of B cells, B-cell subsets, T cells, and NK cells were determined using flow cytometry. Cells were purified, stained with CD19-PerCP-Cy5.5, CD19-PE-Cy7, CD19-APC (all clone SJ25C1), CD45-PerCP (2D1), CD38-PE, CD38-APC, and CD38-PE-Cy7 (HB7), CD27-APC (L128), CD3-PerCP-Cy5.5 (SK7), and CD8-APC-Cy7 (SK1) all from BD Biosciences, polyclonal IgD-FITC, and IgD-PE (all from Southern Biotechnologies, Birmingham, Ala), CD24-FITC (gran-B-ly-1; Sanquin, Amsterdam, The Netherlands), CD4-PE-Cy7 (SFCI12T4D11), and CD45-RA-RD1 (2H4); all from Beckman Coulter, Brea, Calif, and analyzed on a FACSCanto II (BD Biosciences). Data were analyzed with FACSDiva software (BD Biosciences) according to the gating strategy previously published in ref. ^44^. Within the CD45^+^ gate, the following populations were identified: NK cells (CD16^+^CD56^+^), T cells (CD3^+^), CD4^+^ T cells (CD3^+^CD4^+^), CD8^+^ T cells (CD3^+^CD8^+^), B cells (CD19^+^), transitional B cells (CD27^-^IgD^+^CD24^high^CD38^high^), naive mature B cells (CD27^-^IgD^+^CD24^dim^CD38^dim^), marginal zone natural effector B cells (CD27^+^IgD^+^CD38^dim^), and memory B cells (CD27^+^IgD^-^CD38^dim^).

### Measurement of IgGs against vaccine components

Antibody concentrations were measured at the National Institute of Public Health and the Environment (RIVM). IgG antibody concentrations against PT, FHA, PRN, FIM2/3, Dt, tetanus toxin (Tt), Hib PRP, HBsAg, IPV type 1 (Mahoney strain), IPV type 2 (Mef-1 strain), IPV type 3 (Saukett strain), and capsular polysaccharides from pneumococcal serotypes 1, 4, 5, 6B, 7 F, 9 V, 14, 18 C, 19 F, and 23 F were determined by bead-based fluorescent multiplex immunoassays using Luminex xMAP technology^50–53^. FIM2/3, HBsAg, and IPV1-3 concentrations were not measured in the healthy controls. For the *Bordetella pertussis* antigens, the assay was calibrated against the WHO International Pertussis Standard (reference 06/140), interpolated using a 5-parameter fit, and expressed in international units (IU/mL); the pneumococcus assay was calibrated against the international 007sp standard of the NIBSC and expressed in µg/ml, the Hib assay was calibrated against the Hib international standard of NBSC, 09/222 and expressed in µg/ml, and the HepB assay was calibrated against 07/164 of NIBSC and expressed in IU/mL.

The LLOD were 0.21 IU/ml for anti-PT, 0.10 IU/ml for anti-FHA, 0.05 IU/ml for anti-PRN; 0.09 AU/ml for anti-FIM2/FIM3, 0.001 IU/ml for anti-Dt, 0.001 IU/ml for anti-Tt, 0.01 μg/ml for anti-Hib PRP, and 0.005 μg/ml for pneumococcal serotype 1, 0.002 μg/ml for serotype 4, 0.007 μg/ml for serotype 5, 0.004 μg/ml for serotype 6B and 18 C, 0.006 for serotype 7 F and 19 F, 0.003 μg/ml for serotype 9 V and 23 F, and 0.01 μg/ml for serotype 14. Values below the LLOD were imputed with the LLOD. For IPV, the LLOD varied by serotype, and values below the LLOD were imputed as the lowest measured concentration for each: 0.11 IU/mL for IPV1, 0.37 IU/mL for IPV2, and 0.17 IU/mL for IPV3.

The protective thresholds were defined as 0.1 IU/ml for anti-Dt IgG and anti-Tt IgG, 10 IU/ml for anti-HBsAg, 0.15 μg /ml for anti-Hib PRP after primary series and 1.0 μg /ml after booster vaccination, 0.7 IU/mL for anti-IPV1, 2.1 IU/mL for anti-IPV2, 0.6 IU/mL for anti-IPV3, and 0.35 μg /ml for all the anti-capsular IgG against pneumococcal serotypes at as previously described in ref. ^54^. For the response to pertussis vaccination, no well-established correlates for protection are currently available.

### Outcomes

Primary outcome was the response to primary vaccination series and booster vaccination, measured as the proportion of infants with protective IgG concentrations against Dt, Tt, HBsAg, Hib PRP, IPV1-3, and all PCV10 serotypes and GMCs of IgG against PT, FHA, PRN, FIM2/3, Dt, Tt, Hib PRP, IPV1-3, and all PCV10 serotypes. Secondary outcomes included leukocyte counts, T- and B-cell subsets, immunoglobulin concentrations, and infection rate during the first year of life.

### Statistical analysis

The estimated marginal GMCs and 95% CIs were calculated for IgG antibodies against all vaccine components by means of a linear regression analysis. To obtain the GMC, dependent variables were log-transformed^55^. Days after vaccination was included as covariate in all analyses to adjust for time between blood sampling and vaccination. In addition, maternal Tdap vaccination was included as fixed factor in the analyses of pertussis, tetanus and diphtheria antigens to adjust for maternal Tdap status. The contrast between different groups (IBD vs. control anti-TNF versus control, other biological vs control) and estimated marginal GMCs were calculated. In this analysis, covariate value (days after vaccination) was fixed at the group average, whereas fixed factor (maternal Tdap vaccination status) effects were weighed proportionally to the frequency in the dataset to obtain unbiased comparisons. Subgroup analyses were performed to compare GMCs for infants born to Tdap-vaccinated mothers and Tdap-unvaccinated mothers and to compare GMCs between infants of mothers treated with anti-TNF, vedoluzimab, or ustekinumab with control infants. To evaluate the effect of excluding infants who received an additional vaccination at 7 months, sensitivity analyses were performed including these infants. The proportions of infants with IgG concentrations against vaccine components above protective levels were calculated and compared by means of a chi-squared test or Fisher’s exact test. For other outcomes, medians for continuous variables were compared using Mann-Whitney U tests. Proportions of categorical variables were compared by Chi-squared tests or Fisher’s exact tests. Proportions of children below the lower limit of the normal range were compared to the expected proportion (2.5% or 5%) by one-sided binomial tests. Due to the large number of IgG antibodies tested, a Bonferroni adjustment was performed for the primary outcome, assuming 38 tests (one for each antigen (*n* = 19) on two separate timepoints). As such, a two-sided *p*-value < 0.0013 was considered statistically significant. For the secondary outcomes, a *p*-value < 0.05 was considered significant. Analyses were performed by RStudio software, version 4.1.2 by means of the emmeans package and SPSS (version 28). Graphs were generated using RStudio (ggplot2 package) and GraphPad Prism, version 10.2.2. (397).

## Supplementary information

Supplementary material 20-04-2026 - clean.

## Data Availability

The datasets generated and/or analyzed during the current study are not publicly available because time and resources were not available to prepare the data according to the FAIR principles, but the data are available from the corresponding author on reasonable request.
