# Supplementary material for: Response to vaccination and immune status after intrauterine exposure to immunomodulating drugs for maternal IBD
Source: NPJ Vaccines. 2026 Jun 5;11:178. doi: 10.1038/s41541-026-01499-5 (PMC13558638; doi:10.1038/s41541-026-01499-5)
Supplement: Supplementary file 1 [file 41541_2026_1499_MOESM1_ESM.docx]

**Supplementary material**

Supplementary Table 1. Corticosteroid use during pregnancy in mothers with IBD

Supplementary Table 2. Serum concentrations of biologicals in infants born to mothers with IBD

Supplementary Table 3. Baseline characteristics of infants born to IBD mothers and healthy controls for T- and B-cell subset analyses

Supplementary Table 4. GMCs of IgG antibodies against pertussis vaccine components (PT, FHA, PRN), Dt, Tt, Hib PRP, HBsAg, and IPV in infants with intrauterine exposure to immunomodulating drugs and healthy controls

Supplementary Table 5. GMCs of IgG antibodies against pneumococcal conjugate vaccine components in infants with intrauterine exposure to immunomodulating drugs and healthy controls

Supplementary Table 6. GMCs of IgG antibodies against pertussis vaccine components (PT, FHA, PRN), Dt, Tt, Hib PRP, HBsAg, and IPV in infants with intrauterine exposure to anti-TNFα and healthy controls

Supplementary Table 7. GMCs of IgG antibodies against pneumococcal conjugate vaccine components in infants with intrauterine exposure to anti-TNFα and healthy controls

Supplementary Table 8. GMCs of IgG antibodies against pertussis specific vaccine components (PT, FHA, PRN), Dt, and Tt in infants with intrauterine exposure to immunomodulating drugs stratified for maternal Tdap vaccination status

Supplementary Table 9. GMCs of IgG antibodies against pertussis specific vaccine components (PT, FHA, PRN), Dt, and Tt in infants with intrauterine exposure to anti-TNFa, stratified for maternal Tdap vaccination status

Supplementary Table 10. Leukocyte counts in infants born to mothers with IBD

Supplementary Table 11. Lymphocyte subsets in infants with intrauterine exposure to immunomodulating drugs compared to healthy controls

Supplementary Table 12. Proportion of infants with lymphocyte counts below the 5th percentile of the reference range

Supplementary Table 13. Immunoglobulin concentrations in infants born to mothers with IBD

Supplementary Table 14. Infections leading to hospitalization in the first year of life in infants born to mothers with IBD

Supplementary Table 15. Vaccination schedule IBD cohort

Supplementary Table 16. Vaccination schedule control cohort

Supplementary Figure 1. Lymphocyte subsets of infants with intrauterine exposure to immunomodulating drugs compared to healthy controls

**Supplementary Table 1. Corticosteroid use during pregnancy in mothers with IBD^a^**

|  | **Prednisone (oral)** | **Budesonide (oral)** | **Budesonide (rectal)** | **Budesonide (unknown route)** |
| --- | --- | --- | --- | --- |
|  | n=10 | n=10 | n=2 | n=1 |
| Dose (mg/day), median (IQR) | 15 (10-40) | 7.5 (3.0-9.0) | 2 (N/A) | N/A |
| Daily dose ≥10 mg, n (%) | 9 (90) | N/A | N/A | N/A |
| Gestational age at last use, median (IQR) | 37 (21-38) | 39 (37-39) | 39 (N/A) | N/A |
| Continued until delivery, n (%) | 6 (60) | 10 (100) | 1 (50) | N/A |

a Corticosteroid dosing information in 22 mothers with IBD who used corticosteroids during pregnancy. One mother used both prednisone and budesonide.

Abbreviations: IBD, inflammatory bowel disease; IQR, inter quartile range; N/A, not applicable

**Supplementary Table 2. Serum concentrations of biologicals in infants born to mothers with IBD**

|  | **IBD cohort**  **n=137** | |
| --- | --- | --- |
| **Biological^a^** | **Median µg/ml (IQR)** | **Number with detectable concentrations, n (%)** |
| **Mother at delivery** |  |  |
| Adalimumab | 2.7 (1.1 - 7.0) | 28 (90.3) |
| Infliximab | 4.35 (0.75 - 10.93) | 25 (89.3) |
| Vedolizumab | 10.00 (1.40 - 16.75) | 2 (10) |
| Ustekinumab | 3.20 (1.10 - 5.00) | 18 (90) |
| **Infant at delivery** |  |  |
| Adalimumab | 4.8 (2.65 - 7.30) | 30 (93.8) |
| Infliximab | 12.00 (2.70 - 21.00) | 33 (100) |
| Vedolizumab | 4.90 (3.30 - 8.20) | 23 (100) |
| Ustekinumab | 4.10 (2.85 - 6.60) | 24 (100) |
| **Ratio infant vs mother at delivery** |  |  |
| Adalimumab | 1.60 (0.99 - 2.56) | n/a |
| Infliximab | 2.92 (2.11 - 3.96) | n/a |
| Vedolizumab | 0.75 (0.41 - 1.04) | n/a |
| Ustekinumab | 1.81 (1.11 - 2.77) | n/a |
| **Infant at 2 months** |  |  |
| Adalimumab | 1.55 (0.73 - 2.00) | 30 (93.8) |
| Infliximab | 4.10 (0.95 - 6.05) | 33 (100) |
| Vedolizumab | 0.30 (0.10 - 2.00) | 18 (81.8) |
| Ustekinumab | 0.75 (0.48 - 1.48) | 22 (100) |
| **Infant at 6 months** |  |  |
| Adalimumab | 0.05 (0.02 - 0.10) | 25 (78.1) |
| Infliximab | 0.10 (0.03 - 0.20) | 23 (67.6) |
| Vedolizumab | 0.01 (0.01 - 0.01) | 2 (9.1) |
| Ustekinumab | 0.01 (0.01 - 0.02) | 14 (60.9) |
| **Infant at 12 months** |  |  |
| Adalimumab | 0.01 (0.01 - 0.01) | 0 (0) |
| Infliximab | 0.03 (0.03 - 0.20) | 0 (0) |
| Vedolizumab | 0.51 (0.01 - n/a) | 0 (0) |
| Ustekinumab | 0.01 (0.01 - 0.01) | 0 (0) |

^a^The lower limits of detection (LLOD) of the ELISAs were 0.03 µg/ml for infliximab, 0.01 µg/ml for adalimumab, 0.005 µg/ml for golimumab, 0.005 µg/ml for ustekinumab, and 0.01 µg/ml for vedolizumab. In cases where blood sample volumes were insufficient for further dilutions, the LLODs were adjusted. At delivery, the LLOD was increased to 0.1 µg/ml for adalimumab in one mother and one infant, and to 0.3 µg/ml for infliximab in one mother. At 2mo, LLOD was adjusted to 0.2-2.0 µg/ml for vedolizumab in four infants. At 6mo, LLOD was adjusted to 0.08-0.2 µg/ml for adalimumab in three infants, to 0.2 µg/ml for infliximab in two infants, to 0.08-1.0 µg/m for vedolizumab in three infants, and to 0.04 µg/ml for ustekinumab in one infant. At 12mo, the LLOD was adjusted to 0.2-0.3 µg/ml for infliximab in seven infants and to 1.0 µg/ml for vedoluzimab in one infant.
Abbreviations: IQR, interquartile range; IBD, inflammatory bowel disease

**Supplementary Table 3. Baseline characteristics of infants born to IBD mothers and healthy controls for T- and B-cell subset analyses^a^**

|  | **Control cohort**  **n=50** | **IBD cohort**  **n=137** | **p value**  **IBD vs control** |
| --- | --- | --- | --- |
|  |  |  |  |
| Sex, male, n (%) | 26 (52.0 %) | 69 (50.4%) | .87 |
| **2 months** | n/a | n=129 |  |
| Age (mo) at 2mo time point, median (IQR) | n/a | 1.8 (1.6 - 1.9) | n/a |
| **6 months** | n=11 | n=130 |  |
| Age (mo) at 6mo time point, median (IQR) | 6.0 (6.0 - 7.2) | 6.3 (6.0 - 6.5) | .51 |
| **12 months** | n=14 | n=129 |  |
| Age (mo) at 12mo time point, median (IQR) | 12.0 (10.5 - 15.9) | 12.2 (12.0 - 12.6) | .48 |

a Baseline characteristics of 137 infants with intrauterine exposure to immunomodulating drugs and 50 healthy control infants.
Abbreviations: IBD, inflammatory bowel disease; mo, months; n/a, not applicable.

**Supplementary Table 4. GMCs of IgG antibodies against pertussis vaccine components (PT, FHA, PRN), Dt, Tt, Hib PRP, HBsAg, and IPV in infants with intrauterine exposure to immunomodulating drugs and healthy controls^a^**

| **Antigen** | **Healthy controls** | **IBD cohort** | **p value** |
| --- | --- | --- | --- |
| **6 months** | **n=103** | **n=137** |  |
| PT | 44.1 (36.9-52.6) | 54.4 (46.6-63.7) | .08 |
| FHA | 40.8 (35.7-46.6) | 36.8 (32.8-41.4) | .26 |
| PRN | 36.6 (29.3-45.7) | 48.5 (40.1-58.6) | .07 |
| FIM2/3 | n/a | 183 (160-209) | n/a |
| Dt | 0.18 (0.14-0.22) | 0.25 (0.21-0.31) | .02 |
| Tt | 0.83 (0.71-0.97) | 3.74 (3.26-4.28) | <.001 |
| Hib PRP | 0.30 (0.22-0.41) | 4.30 (3.30-5.60) | <.001 |
| HBsAg | n/a | 6675.0 (5661.7-7869.8) | n/a |
| IPV1 | n/a | 13.5 (10.7-17.0) | n/a |
| IPV2 | n/a | 27.1 (21.2-34.7) | n/a |
| IPV3 | n/a | 6.56 (5.17-8.33) | n/a |
| **12 months^b^** | **n=103** | **n=119** |  |
| PT | 93.4 (78.8-111.0) | 120.8 (102.7-142.0) | .03 |
| FHA | 80.6 (69.3-93.8) | 49.2 (42.5-56.9) | <.001 |
| PRN | 108 (86.5-134) | 129 (105.3-158) | .26 |
| FIM2/3 | n/a | 369 (319-426) | n/a |
| Dt | 0.92 (0.78-1.08) | 1.16 (1.00-1.36) | .04 |
| Tt | 4.04 (3.55-4.59) | 10.25 (9.07-11.58) | <.001 |
| Hib PRP | 11.25 (8.62-14.68) | 5.38 (4.23-6.85) | <.001 |
| HBsAg | n/a | 9087.7 (7295.4-11320.4) | n/a |
| IPV1 | n/a | 32.7 (28.7-37.3) | n/a |
| IPV2 | n/a | 65.6 (56.6-75.9) | n/a |
| IPV3 | n/a | 14.4 (12.2-17.0) | n/a |

a Data are presented as geometric means (95% CI) in IU/mL (Pt, FHA, PRN, Dt, TT, HBsAg, IPV) and μg/ml (Hib-PRP).
b At 12 months, 7 patients were excluded from the IBD group because of an extra DTaP-IPV-Hib-HepB vaccination at 7 months.

Abbreviations: GMC, Geometrical mean concentration; IBD, inflammatory bowel disease; PT, pertussis toxin; FHA, filamentous haemagglutinin; PRN, pertactin; Dt diphtheria toxoid; Tt, tetanus toxin; Hib PRP, H. influenzae type b; HBsAg, Hepatitis B; IPV, inactivated polio virus; n/a, not applicable.

**Supplementary Table 5. GMCs of IgG antibodies against pneumococcal conjugate vaccine components in infants with intrauterine exposure to immunomodulating drugs and healthy controls^a^**

| **Antigen** | **Healthy controls** | **IBD cohort** | **p value** |
| --- | --- | --- | --- |
| **6 months** | **n=102** | **n=137** |  |
| Ps 1 | 2.71 (2.51-3.43) | 2.70 (2.21-3.30) | .98 |
| Ps 4 | 0.61 (0.49-0.76) | 2.41 (2.00-2.89) | <.001 |
| Ps 5 | 1.62 (1.29-2.03) | 3.36 (2.76-4.08) | <.001 |
| Ps 6B | 0.33 (0.22-0.48) | 0.46 (0.33-0.63) | .21 |
| Ps 7F | 3.80 (3.17-4.56) | 4.13 (3.54-4.84) | .50 |
| Ps 9V | 1.58 (1.29-1.92) | 1.90 (1.61-2.26) | .16 |
| Ps 14 | 2.76 (2.18-3.50) | 3.00 (2.45-3.68) | .61 |
| Ps 18C | 0.93 (0.71-1.23) | 4.93 (3.90-6.24) | <.001 |
| Ps 19F | 7.31 (5.49-9.74) | 5.84 (4.57-7.48) | .25 |
| Ps 23F | 0.54 (0.40-0.72) | 0.58 (0.45-0.74) | .72 |
| **12 months^b^** | **n=98** | **n=109** |  |
| Ps 1 | 9.55 (7.88-11.58) | 9.22 (7.69-11.07) | .80 |
| Ps 4 | 1.78 (1.49-2.12) | 7.08 (5.99-8.37) | <.001 |
| Ps 5 | 5.42 (4.54-6.48) | 13.77 (11.63-16.31) | <.001 |
| Ps 6B | 5.09 (4.31-6.01) | 6.09 (5.21-7.13) | .12 |
| Ps 7F | 9.77 (8.34-11.44) | 11.93 (10.27-13.86) | .07 |
| Ps 9V | 5.77 (4.98-6.69) | 6.69 (5.82-7.70) | .15 |
| Ps 14 | 4.72 (3.78-5.89) | 6.76 (5.48-8.33) | .02 |
| Ps 18C | 7.79 (6.44-9.43) | 18.58 (15.51-22.24) | <.001 |
| Ps 19F | 35.60 (30.02-42.22) | 14.08 (11.98-16.55) | <.001 |
| Ps 23F | 3.84 (3.19-4.62) | 4.07 (3.42-4.86) | .65 |

a Data are presented as geometric means (95% CI) in µg/mL.
b At 12 months, 17 patients were excluded from the IBD group because of an extra pneumococcal vaccination at age 7 months

Abbreviations: GMC, geometrical mean concentration; IBD, inflammatory bowel disease; Ps, pneumococcal serotype.

**Supplementary 6. GMCs of IgG antibodies against pertussis vaccine components (PT, FHA, PRN), Dt, Tt, Hib PRP, HBsAg, and IPV in infants with intrauterine exposure to anti-TNFα and healthy controls^a^**

| **Antigen** | **healthy controls** | **IBD TNFα** | **p value** |
| --- | --- | --- | --- |
| **6 months** | **n=103** | **n=69** |  |
| PT | 47.6 (40.2-56.3) | 59.8 (47.8-74.8) | .16 |
| FHA | 44.2 (38.7-50.4) | 37.9 (31.8-45.1) | .171 |
| PRN | 38.4 (30.9-47.6) | 45.8 (35.1-59.8) | .32 |
| FIM2/3 | n/a | 179 (147-218) | n/a |
| Dt | 0.19 (0.15-0.24) | 0.35 (0.27-0.45) | .001 |
| Tt | 0.84 (0.72-0.98) | 4.03 (3.30-4.92) | <.001 |
| Hib PRP | 0.30 (0.22-0.41) | 4.35 (2.99-6.34) | <.001 |
| HBsAg | n/a | 6859.7 (5534.6-8502.0) | n/a |
| IPV1 | n/a | 11.6 (8.15-16.4) | n/a |
| IPV2 | n/a | 22.0 (14.9-32.5) | n/a |
| IPV3 | n/a | 6.07 (4.32-8.53) | n/a |
| **12 months^b^** | **n=103** | **n=60** |  |
| PT | 99.8 (85.2-117) | 121.4 (97.1-152) | .16 |
| FHA | 84.5 (73.0-97.8) | 51.8 (42.1-63.8) | <.001 |
| PRN | 109 (88.1-136) | 125 (93.3-166) | .49 |
| FIM2/3 | n/a | 356 (299-424) | n/a |
| Dt | 0.95 (0.82-1.10) | 1.36 (1.10-1.70) | .008 |
| Tt | 4.05 (3.58-4.58) | 9.92 (8.32-11.84) | <.001 |
| Hib PRP | 11.31 (8.57-14.92) | 5.33 (3.73-7.60) | .001 |
| HBsAg | n/a | 9429.8 (7132.4-12467.2) | n/a |
| IPV1 | n/a | 30.7 (25.8-36.6) | n/a |
| IPV2 | n/a | 63.2 (51.7-77.3) | n/a |
| IPV3 | n/a | 12.8 (9.97-16.3) | n/a |

a Data are presented as geometric means (95% CI) in IU/mL (Pt, FHA, PRN, Dt, TT, HBsAg, IPV) and μg/ml (Hib-PRP).
b At 12 months, 3 patients were excluded from the IBD TNF**α** group because of an extra DTaP-IPV-Hib-HepB vaccination at 7 months.

Abbreviations: GMC. Geometrical mean concentration; IBD. inflammatory bowel disease; anti-TNFα. anti-Tumor Necrosis Factor alpha; PT, pertussis toxin; FHA, filamentous haemagglutinin; PRN, pertactin; Dt diphtheria toxoid; TT, tetanus toxin; Hib PRP, H. influenzae type b; HBsAg, Hepatitis B; IPV, inactivated polio virus; n/a, not applicable

**Supplementary Table 7. GMCs of IgG antibodies against pneumococcal conjugate vaccine components in infants with intrauterine exposure to anti-TNFα and healthy controls^a^**

| **Antigen** | **Healthy controls** | **IBD TNFa** | **p value** |
| --- | --- | --- | --- |
| **6 months** | **n=102** | **n=69** |  |
| Ps 1 | 2.71 (2.14-3.44) | 2.42 (1.81-3.24) | .55 |
| Ps 4 | 0.61 (0.49-0.76) | 2.20 (1.69-2.87) | <.001 |
| Ps 5 | 1.61 (1.29-2.02) | 2.89 (2.20-3.81) | .002 |
| Ps 6B | 0.33 (0.22-0.48) | 0.32 | .92 |
| Ps 7F | 3.77 (3.11-4.56) | 3.55 (2.81-4.49) | .71 |
| Ps 9V | 1.57 (1.28-1.93) | 1.77 (1.38-2.27) | .46 |
| Ps 14 | 2.73 (2.15-3.48) | 2.60 (1.95-3.48) | .80 |
| Ps 18C | 0.95 (0.72-1.25) | 4.55 (3.24-6.39) | <.001 |
| Ps 19F | 7.31 (5.43-9.86) | 7.22 (5.02-10.39) | .96 |
| Ps 23F | 0.55 (0.40-0.74) | 0.52 (0.36-0.74) | .81 |
| **12 months^*^** | **n=98** | **n=54** |  |
| Ps 1 | 99.66 (8.00-11.65) | 7.99 (6.20-10.30) | .24 |
| Ps 4 | 1.79 (1.52-2.10) | 6.39 (5.15-7.94) | <.001 |
| Ps 5 | 5.46 (4.58-6.50) | 11.76 (9.28-14.89) | <.001 |
| Ps 6B | 5.10 (4.32-6.03) | 5.56 (4.43-6.98) | .55 |
| Ps 7F | 9.83 (8.46-11.43) | 10.15 (8.29-12.44) | .80 |
| Ps 9V | 5.83 (5.07-6.17) | 5.75 (4.76-6.95) | .91 |
| Ps 14 | 4.75 (3.77-5.97) | 5.58 (4.10-7.61) | .41 |
| Ps 18C | 7.78 (6.55-9.24) | 17.33 (13.76-21.86) | <.001 |
| Ps 19F | 35.65 (29.76-42.72) | 14.06 (11.01-17.96) | <.001 |
| Ps 23F | 0.55 (0.40-0.74) | 3.55 (2.80-4.51) | .56 |

a Data are presented as geometric means (95% CI) in µg/mL.
b At 12 months, 9 patients were excluded from the IBD TNFα group because of an extra pneumococcal vaccination at age 7 months
Abbreviations: GMC, Geometrical mean concentration; IBD, inflammatory bowel disease; Ps, pneumococcal serotype.

**Supplementary Table 8. GMCs of IgG antibodies against pertussis specific vaccine components (PT, FHA, PRN), Dt, and Tt in infants with intrauterine exposure to immunomodulating drugs stratified for maternal Tdap vaccination status^a^**

| **Antigen** | **IBD any drugs Tdap+** | **IBD any drugs Tdap-** | **p value** |
| --- | --- | --- | --- |
| **6 months** | **n=119** | **n=18** |  |
| PT | 43.54 (37.36-50.73) | 95.83 (64.80-141.74) | <.001 |
| FHA | 29.18 (26.03-32.70) | 66.29 (49.51-88.76) | <.001 |
| PRN | 41.66 (34.26-50.65) | 63.38 (38.43 - 104.5) | .12 |
| FIM2/3 | 170.54 (148.03-196.47) | 293.14 (203-421.84) | .04 |
| Dt | 0.19 (0.15-0.23) | 0.52 (0.31-0.86) | <.001 |
| Tt | 3.72 (3.25-4.25) | 3.78 (2.69-5.32) | .92 |
| **12 months*** | **n=102** | **n=17** |  |
| PT | 105.70 (90.00-124.14) | 164.60 (111.33-243.35) | .04 |
| FHA | 43.30 (37.51-49.98) | 66.03 (46.58-93.60) | .03 |
| PRN | 128.97 (104.30-159.48) | 104.44 (62.32-175.02) | .46 |
| FIM2/3 | 366.59 (313.64-428.49) | 381.32 (260.05-559.14) | .06 |
| Dt | 1.03 (0.88-1.20) | 1.55 (1.07-2.25) | .05 |
| Tt | 11.69 (10.36-13.20) | 7.55 (5.63-10.13) | .007 |

a Data are presented as geometric means (95% CI) in IU/mL.
Tdap+, maternal Tdap vaccination during pregnancy; Tdap-, no maternal Tdap vaccination during pregnancy
Abbreviations: GMC, Geometrical mean concentration; IBD, inflammatory bowel disease; Tdap, tetanus, diphtheria, and acellular pertussis vaccination; PT, pertussis toxin; FHA, filamentous haemagglutinin; PRN, pertactin; Dt diphtheria toxoid; Tt, tetanus toxin.

**Supplementary Table 9. GMCs of IgG antibodies against pertussis specific vaccine components (PT, FHA, PRN), Dt, and Tt in infants with intrauterine exposure to Anti-TNFa, stratified for maternal Tdap vaccination status^a^**

| **Antigen** | **IBD TNFa Tdap+** | **IBD TNFa Tdap-** | **p value** |
| --- | --- | --- | --- |
| **6 months** | **n=57** | **n=12** |  |
| PT | 46.16 (36.99-57.59) | 94.72 (58.74-152.75) | .008 |
| FHA | 29.82 (25.10-35.44) | 57.98 (39.94-84.18) | .002 |
| PRN | 37.04 (27.87-49.22) | 60.36 (32.66-111.56) | .16 |
| FIM2/3 | 155.04 (124.89-192.46) | 351.37 (219.32-562.93) | .003 |
| Dt | 0.25 (0.19-0.34) | 0.57 (0.31-1.07) | .02 |
| Tt | 3.80 (3.12-4.64) | 4.46 (2.91-6.84) | .51 |
| **12 months** | **n=49** | **n=11** |  |
| PT | 104.47 (83.41-130.85) | 156.14 (97.60-249.80) | .13 |
| FHA | 47.38 (38.44-58.41) | 60.26 (38.93-93.26) | .33 |
| PRN | 122.36 (89.53-167.23) | 102.41 (53.36-196.53) | .63 |
| FIM2/3 | 331.63 (273.45-402.19) | 488.62 (324.19-736.46) | .09 |
| Dt | 1.22 (0.98-1.51) | 1.65 (1.05-2.59) | .24 |
| Tt | 10.61 (8.88-12.67) | 8.88 (6.13-12.86) | .40 |

a Data are presented as geometric means (95% CI) in IU/mL.

Tdap+, maternal Tdap vaccination during pregnancy; Tdap-, no maternal Tdap vaccination during pregnancy
Abbreviations: GMC, Geometrical mean concentration; IBD, inflammatory bowel disease; Tdap, tetanus, diphtheria, and acellular pertussis vaccination; anti-TNFα, anti-Tumor Necrosis Factor alpha; PT, pertussis toxin; FHA, filamentous haemagglutinin; PRN, pertactin; Dt, diphtheria toxoid; Tt, tetanus toxin; Hib PRP, H. influenzae type b; HBsAg, Hepatitis B.

**Supplementary Table 10. Leukocyte counts in infants born to mothers with IBD^a^**

|  | **Reference values^b^** | **IBD cohort**  **n=137** | |  |
| --- | --- | --- | --- | --- |
| **Cells/µL** | **(2.5th - 97.5th percentile)** | **median (2.5th - 97.5th percentile)** | **Infants with numbers below 2.5^th^ percentile of reference value, n (%)** | **p value^c^** |
| **Cord blood** |  | **n=130** |  |  |
| Leukocytes | 8000 - 15400 | 19000 (9048 - 35000) | 1 (0.8) | .16 |
| Thrombocytes (x10^3^) | 144 - 449 | 272 (106 - 409) | 6 (4.7) | .10 |
| Lymphocytes | 640 - 7080 | 4860 (1681 - 9299) | 1 (0.9) | .25 |
| Monocytes | 320 - 2930 | 1645 (401 - 3977) | 2 (2.0) | .54 |
| Neutrophils | 1680 - 8470 | 10990 (2413 - 25591) | 0 (0) | .07 |
| **2 months** |  | **n=127** |  |  |
| Leukocytes | 6000 - 15000 | 9400 (5853 - 16183) | 3 (2.4) | .61 |
| Thrombocytes (x10^3^) | 229 - 597 | 471 (219 - 755) | 3 (2.5) | .64 |
| Lymphocytes | 720 - 10200 | 6835 (3998 - 13088) | 0 (0) | .06 |
| Monocytes | 240 - 2550 | 620 (76 - 2116) | 18 (16.2) | <.001 |
| Neutrophils | 900 - 9000 | 1720 (772 - 4196) | 7 (5.1) | .06 |
| **6 months** |  | **n=134** |  |  |
| Leukocytes | 6000 - 15000 | 11000 (6135 - 18935) | 2 (1.5) | .35 |
| Thrombocytes (x10^3^) | 229 - 597 | 412 (248 - 686) | 1 (0.8) | .17 |
| Lymphocytes | 720 - 10200 | 7300 (3161 - 13759) | 0 (0) | .05 |
| Monocytes | 240 - 2550 | 600 (81 - 1874) | 12 (9.9) | <.001 |
| Neutrophils | 900 - 9000 | 2400 (941 - 7186) | 2 (1.5) | .37 |
| **12 months** |  | **n=125** |  |  |
| Leukocytes | 6000 - 13500 | 9900 (4890 - 18465) | 6 (4.8) | .09 |
| Thrombocytes (x10^3^) | 206 - 459 | 365 (186 - 725) | 4 (3.1) | .39 |
| Lymphocytes | 780 - 9450 | 6470 (2486 - 11544) | 0 (0) | .05 |
| Monocytes | 240 -1350 | 645 (49 - 1432) | 12 (10.3) | <.001 |
| Neutrophils | 1380 - 9320 | 2760 (942 - 6680) | 12 (9.8) | <.001 |

SI conversion factors: To convert leukocytes, lymphocytes, monocytes, and neutrophils to cells x10^9^/L multiply by 0.001; thrombocytes to cells x10^9^/L multiply by 1.
a Data are displayed as medians (2.5 - 97.5 percentile) and as number of infants with values below the 2.5^th^ percentile of the reference value.
b Obtained from the reference guide of the Erasmus MC, the Netherlands: www.erasmusmc.nl/nl-nl/laboratoriumdiagnostiek/bepalingenwijzer [accessed June 2025]
c P values are derived from one sample binomial tests, comparing the observed percentage of children with leukocyte counts below the 2.5 percentile of the reference value to the expected percentage (2.5).
Abbreviation: IBD, Inflammatory bowel disease

**Supplementary Table 11. Lymphocyte subsets in infants with intrauterine exposure to immunomodulating drugs compared to healthy controls^a^**

| cells/µL | **Healthy controls^b^** | **IBD cohort** | **p value** |
| --- | --- | --- | --- |
| **Cord blood** | **n=25** | **n=123** |  |
| T cells | 2900 (2265 - 3395) | 3160 (2230 - 4320) | .14 |
| CD4+ T cells | n/a | 2090 (1530 - 2910) | n/a |
| CD8+ T cells | n/a | 840 (640 - 1220) | n/a |
| B cells | 590 (355 - 760) | 760 (470 - 1070) | .02 |
| NK cells | 200 (135 - 355) | 940 (420 - 1410) | <.001 |
| Transitional B cells | 157.68 (87.36 - 198.98) | 197.00 (89.00 - 316.00) | .06 |
| Naive mature B cells | 377.40 (206.79 - 482.56) | 495.00 (284.00 - 743.00) | .03 |
| MZ natural effector B cells | 30.09 (18.28 - 49.36) | 40.00 (23.00 - 73.00) | .03 |
| Memory B cells | 2.45 (1.09 - 7.56) | 2.00 (1.00 - 4.00) | .07 |
| **2 months** | n/a | **n=129** |  |
| T cells | n/a | 4850 (3920 - 5770) | n/a |
| CD4+ T cells | n/a | 3590 (2840 - 4315) | n/a |
| CD8+ T cells | n/a | 1080 (900 - 1355) | n/a |
| B cells | n/a | 1510 (1085 - 2070) | n/a |
| NK cells | n/a | 360 (204 - 510) | n/a |
| Transitional B cells | n/a | 425.00 (310.00 - 619.00) | n/a |
| Naive mature B cells | n/a | 944 (652.75 - 1285.50) | n/a |
| MZ natural effector B cells | n/a | 64.00 (39.25 - 100.25) | n/a |
| Memory B cells | n/a | 5.00 (3.00 - 7.00) | n/a |
| **6 months** | **n=11** | **n=130** |  |
| T cells | 3420 (2180 - 4300) | 4940 (3698 - 6013) | .002 |
| CD4+ T cells | n/a | 3460 (2688 - 4263) | n/a |
| CD8+ T cells | n/a | 1135 (913 - 1493) | n/a |
| B cells | 1580 (1150 - 1850) | 1750 (1330 - 2393) | .14 |
| NK cells | 380 (220 - 560) | 330 (228 - 473) | .55 |
| Transitional B cells | 369.60 (186.30 - 397.21) | 388.50 (256.75 - 545.00) | .22 |
| Naive mature B cells | 1023.64 (761.40 - 1326.78) | 1330.50 (874.50 - 1772.25) | .10 |
| MZ natural effector B cells | 86.35 (39.96 - 129.50) | 91.00 (59.75 - 133.00) | .45 |
| Memory B cells | 23.1 (12.0 - 40.47) | 18.50 (13.00 - 25.25) | .32 |
| **12 months** | **n=14** | **n=129** |  |
| T cells | 3365 (2688 - 3620) | 4280 (3365 - 5120) | .008 |
| CD4+ T cells | n/a | 2930 (2235 - 3545) | n/a |
| CD8+ T cells | n/a | 1060 (785 - 1465) | n/a |
| B cells | 1330 (1028 - 1795) | 1510 (1170 - 2070) | .35 |
| NK cells | 425 (270 - 625) | 320 (210 - 460) | .15 |
| Transitional B cells | 202.07 (160.70 - 288.89) | 276.00 (194.00 - 410.00) | .05 |
| Naive mature B cells | 979.30 (738.39 - 1333.69) | 1015.00 (709.50 - 1421.50) | .75 |
| MZ natural effector B cells | 74.97 (54.07 - 126.33) | 107.00 (71.00 - 154.50) | .13 |
| Memory B cells | 52.66 (26.73 - 69.31) | 35.00 (25.00 - 50.00) | .24 |

SI conversion factor: To convert to cellsx10^9^/L multiply by 0.001
a Data are presented as median absolute numbers (IQR). T cells CD3+, B cells CD19+, natural killer (NK) cells CD3-CD16/56+, transitional B cells CD38high/CD24high, naive mature B cells CD38dim/CD24dim/IgD+/CD27-, MZ/natural effector B cells CD38dim/IgD+/CD27+, memory B cells CD38dim/IgD-/CD27+.
b healthy controls from a previously recruited cohort (Driessen et al., 2011)
Abbreviations: IBD, inflammatory bowel disease; n/a, not applicable
 **Supplementary Table 12. Proportion of infants with lymphocyte counts below the 5th percentile of the reference range**

| **cells/µL** | **Reference range^a^**  **median (p5 -p95)** | **IBD cohort: infants below 5^th^ percentile of reference range**  **n (%)** | **p value^b^** |
| --- | --- | --- | --- |
| **Cord blood** | **n=25** | **n=123** |  |
| T cells | 2900 (1228 - 3780) | 5 (4.1%) | .42 |
| B cells | 590 (172 - 1266) | 10 (8.1%) | .09 |
| NK cells | 200 (72 - 669) | 4 (3.3%) | .26 |
| Transitional B cells | 157.68 (28.61 - 274.44) | 8 (6.7%) | .25 |
| Naive mature B cells | 377.40 (120.10 - 828.43) | 8 (6.7%) | .25 |
| MZ natural effector B cells | 30.09 (3.51 - 70.37) | 6 (5.0%) | .61 |
| Memory B cells | 2.45 (0.31 - 23.27) | 16 (13.3%) | <.001 |
| **6 months** | **n=11** | **n=130** |  |
| T cells | 3420 (2000 - n/a) | 2 (1.5%) | .04 |
| B cells | 1580 (650 - n/a) | 2 (1.5%) | .04 |
| NK cells | 380 (130 - n/a) | 4 (3.1%) | .22 |
| Transitional B cells | 369.60 (170.20 - n/a) | 7 (5.6%) | .44 |
| Naive mature B cells | 1023.64 (417.30 - n/a) | 1 (0.8%) | .01 |
| MZ natural effector B cells | 86.35 (29.9 - n/a) | 2 (1.6%) | .05 |
| Memory B cells | 28.40 (9.75 - n/a) | 14 (11.1%) | .004 |
| **12 months** | **n=14** | **n=129** |  |
| T cells | 3365 (1940 - n/a) | 5 (3.8%) | .37 |
| B cells | 1330 (770 - n/a) | 6 (4.7%) | .53 |
| NK cells | 425 (150 - n/a) | 13 (10.1%) | .01 |
| Transitional B cells | 202.07 (53.46 - n/a) | 0 (0%) | .002 |
| Naive mature B cells | 979.30 (458.99 - n/a) | 6 (4.8%) | .56 |
| MZ natural effector B cells | 74.97 (32.34 - n/a) | 4 (3.2%) | .24 |
| Memory B cells | 52.65 (7.9 - n/a) | 1 (0.8%) | .01 |

SI conversion factor: To convert to cellsx10^9^/L multiply by 0.001
a Calculated from a historical healthy control cohort (Driessen et al., 2011)
b P values are derived from one sample binomial tests, comparing the observed percentage of children with lymphocyte counts below the 5^th^ percentile of the reference value to the expected percentage (5.0%)

**Supplementary Table 13. Immunoglobulin concentrations in infants born to mothers with IBD^a^**

|  | **Reference value^b^** | **IBD cohort** | |  |
| --- | --- | --- | --- | --- |
| **mg/dL** | **2.5th-97.5th percentile** | **median (2.5th-97.5th percentile)** | **infants with numbers below 2.5^th^ percentile of reference value,**  **n (%)^d^** | **p value^c^** |
| **2 months** |  | **n=129** |  |  |
| IgM | 10 - 55 | 30 (14 - 70) | 1 (0.8) | .17 |
| IgA | 6 - 57 | 9 (3 - 50) | 32 (28.1) | <.001 |
| IgG | 260 - 780 | 510 (340 - 708) | 1 (0.8) | .17 |
| IgG1 | 180 - 670 | 330 (151 - 549) | 6 (5.0) | .08 |
| IgG2 | 38 - 210 | 110 (53 - 203) | 1 (0.8) | .20 |
| IgG3 | 14 - 70 | 18 (5 - 55) | 36 (30.0) | <.001 |
| IgG4 | 2 - 36 | 7 (1 - 45) | 10 (8.7) | <.001 |
| **6 months** |  | **n=134** |  |  |
| IgM | 17 - 120 | 51 (22 - 1.39) | 0 (0) | .04 |
| IgA | 16 - 98 | 20 (5 - 650) | 52 (41.3) | <.001 |
| IgG | 260 - 1390 | 410 (204 - 825) | 13 (9.8) ^e^ | <.001 |
| IgG1 | 200 - 770 | 301 (109 - 570) | 22 (18.2) | <.001 |
| IgG2 | 34 - 230 | 66 (29 - 146) | 7 (5.8) | .03 |
| IgG3 | 14 - 70 | 31 (8 - 114) | 10 (8.3) | <.001 |
| IgG4 | 1 - 43 | 2 (1 - 23) | 6 (5.4) | .06 |
| **12 months** |  | **n=125** |  |  |
| IgM | 10 - 87 | 75 (30 - 133) | 0 | .04 |
| IgA | 19 - 110 | 33 (5 - 83) | 19 (15.4)^f^ | <.001 |
| IgG | 260 - 1390 | 630 (255 - 1053) | 3 (2.4) | .62 |
| IgG1 | 250 - 820 | 470 (170 - 836) | 8 (7.1) | .008 |
| IgG2 | 38 - 240 | 76 (27 - 175) | 9 (8.0) | .002 |
| IgG3 | 15 - 107 | 37 (14 - 122) | 3 (2.7) | .54 |
| IgG4 | 1 - 62 | 5 (0 - 73) | 5 (4.4) | .15 |

SI conversion factor: To convert to g/L multiply by 0.01
a Data are displayed as medians (2.5^th^ – 97.5^th^ percentile) and as number of infants with counts below the 2.5^th^ percentile of the reference value.
b derived from <https://www.sanquin.org/nl/producten-en-diensten/diagnostiek/diagnostische-testen/index>
c One sided p values are derived from one sample binomial tests, comparing the observed percentage of children with immunoglobulin levels below the 2.5th percentile of the reference value to the expected percentage (2.5).
d Infants with immunoglobulin levels lower than the lower limit of quantification (LLOQ) and higher than the 2.5^th^ percentile of the reference value were excluded from the one sample binomial test. For example, at 2 months, infants with IgA levels <5 mg/dL were excluded. The following number of infants were excluded: at 2 months for IgM 2 infants, IgA 13 infants, IgG 1 infant, IgG4 3 infants; at 6 months for IgA 7 infants, IgG 3 infants, IgG4 8 infants; at 12 months for IgA 1 infants, IgG4 1 infant.
e Mild IgG deficiency ranging from 170-240 mg/dL
f Mostly mild IgA deficiency ranging from 10-18 mg/dL (15 infants). One infant had an IgA concentration of 3 mg/dL.
Abbreviations: IBD, inflammatory bowel disease. IgM, immunoglobulin M. IgA, immunoglobulin A, IgG, immunoglobulin G.

**Supplementary Table 14. Infections leading to hospitalization in the first year of life in infants born to mothers with IBD**

|  | **IBD cohort**  **n=137** |
| --- | --- |
| **Type of infection** | **Number of infants (%)** |
| Respiratory tract infection |  |
| Viral | 6 (4.4) |
| Bacterial | 0 (0) |
| Unknown | 2 (1.5) |
| Urinary tract infection | 2 (1.5) |
| Gastrointestinal infection | 1 (0.7)^a^ |
| Skin and soft tissue | 1 (0.7) |

a one infant with a Medium chain acyl-CoA dehydrogenase (MCAD) deficiency was admitted twice during the first year of life
Abbreviation: IBD, inflammatory bowel disease

**Supplementary Table 15. Vaccination schedule IBD cohort^a^.**

| **Age** | **Injection 1** | **Injection 2** |
| --- | --- | --- |
| 22+ wk gestation (maternal)^b^ | Tdap | - |
| 2 months | DTaP5-IPV-Hib-HepB | - |
| 3 months | DTaP5-IPV-Hib-HepB | PCV10 |
| 5 months | DTaP5-IPV-Hib-HepB | PCV10 |
| 11 months | DTaP5-IPV-Hib-HepB | PCV10 |

a vaccinations administered according to Dutch National Immunization Program, offered by the National Institute for Public Health and the Environment (RIVM), as applicable during the study period (2018-2022). Schedule adapted from [43-45].

b all pregnant women were offered maternal Tdap vaccination as part of the National Immunization Program, however, being non-Tdap vaccinated was no exclusion criteria for study participation.

Abbreviations: wk, weeks; Tdap, tetanus-diphtheria-acellular pertussis vaccine (*Boostrix)*; DTaP5-IPV-Hib-HepB, diphtheria-tetanus-five antigen acellular pertussis-inactivated poliovirus-haemophilus influenza type b-hepatitis B vaccine (*Vaxelis)*, PCV10, 10-valent pneumococcal conjugate vaccine (*Synflorix)*

**Supplementary Table 16. Vaccination schedule control cohort^a^.**

| **Age** | **Injection 1** | **Injection 2** |
| --- | --- | --- |
| 30-32 wk gestation (maternal)^b^ | Tdap | - |
| 2 months | - | - |
| 3 months | DTaP3-IPV-Hib-HepB | PCV10 |
| 5 months | DTaP3-IPV-Hib-HepB | PCV10 |
| 11 months | DTaP3-IPV-Hib-HepB | PCV10 |

a vaccinations administered according to Dutch National Immunization Program, offered by the National Institute for Public Health and the Environment (RIVM), as applicable during the study period of the previously published control cohort (2014-2016) [41]. Schedule adapted from [43-45]. Within the control cohort, maternal Tdap vaccination was not part of the National Immunization Program but was administered as part of the study protocol.

b As part of the study protocol, women either received a Tdap vaccine between weeks 30-32 of pregnancy or within 48 hours after delivery as control [41].

Abbreviations: wk, weeks;Tdap (*Boostrix)*, tetanus-diphtheria-acellular pertussis vaccine; DTaP5-IPV-Hib-HepB, diphtheria-tetanus-three antigen acellular pertussis-inactivated poliovirus-haemophilus influenza type b-hepatitis B vaccine (*Infanrix Hexa)*; PCV10, 10-valent pneumococcal conjugate vaccine (*Synflorix)*

**Suppelementary Figure 1. Lymphocyte subsets of infants with intrauterine exposure to immunomodulating drugs compared to healthy controls**

The dots represent individual values. The lines with error bars represent the median and IQR. T cells CD3^+^, B cells CD19^+^, natural killer (NK) cells CD3^-^CD16/56^+^, transitional B cells CD38^high^/CD24^high^, naive mature B cells CD38^dim^/CD24^dim^/IgD^+^/CD27^-^, MZ/natural effector B cells CD38^dim^/IgD^+^/CD27^+^, memory B cells CD38^dim^/IgD^-^/CD27^+^.
Abbreviations: IBD, inflammatory bowel disease; Ns, non-significant; *p<0.05; **p<0.01; ***p<0.001.
